# Genetic analysis of resistance to *Pseudomonas syringae* pv*. actinidiae* (Psa) in a kiwifruit progeny test: an application of generalised linear mixed models (GLMMs)

**DOI:** 10.1186/2193-1801-3-547

**Published:** 2014-09-22

**Authors:** Nihal H De Silva, Luis Gea, Russell Lowe

**Affiliations:** The New Zealand Institute for Plant & Food Research Limited (PFR), Mt Albert Research Centre, 120 Mt Albert Road, Auckland, 1142 New Zealand; PFR, Te Puke Research Centre, 412 No. 1 Road, RD 2, Te Puke, 3182 New Zealand

**Keywords:** Kiwifruit, Tetraploid, Psa resistance, Heritability, GLMM, SAS

## Abstract

Linear Mixed models (LMMs) that incorporate genetic and spatial covariance structures have been used for many years to estimate genetic parameters and to predict breeding values in animal and plant breeding. Although the theoretical aspects for extending LMM to generalised linear mixed models (GLMMs) have been around for some time, suitable software has been developed only within the last decade or so. The GLIMMIX procedure in SAS® is becoming popular for fitting GLMMs in various disciplines. Applications of GLMMs to genetic analysis have been limited, probably because of the complexity of the models used. This is particularly so for Proc GLIMMIX because, unlike ASReml software, it is not specifically tailored for analysis of breeding data and some pre-procedure coding is necessary. Binary data that fits the GLMM framework is commonly encountered in breeding experiments, such as when evaluating individuals for resistance by observing the presence or absence of disease. Bacterial canker (Psa) caused by *Pseudomonas syringae* pv. *actinidiae* is a serious disease of kiwifruit in New Zealand and other kiwifruit-producing countries. Data from a progeny test trial was available to identify parents with high breeding values for resistance. We successfully applied the GLIMMIX procedure for this purpose. Heritability for resistance was moderate, and we identified two parents and their family as having high potential for Psa resistance breeding. There are several potential pitfalls when using GLMMs with binary data and these are briefly discussed.

## Introduction

*Pseudomonas syringae* pv. *actinidiae* (Psa) is a pathogenic bacterium of kiwifruit (*Actinidia* spp.). The virulent form Psa-V is now well established in New Zealand following its first detection there in November 2010 (Everett et al. 2011). From a biological perspective, Psa-V is the causal agent of a number of disease symptoms, including leaf spots and necrosis, flower wilting, cane dieback, and branch and trunk cankers, often leading to vine death in the case of susceptible cultivars (http://www.kvh.org.nz/). The disease has been very damaging to the New Zealand kiwifruit industry: the cost in net present value is expected to be between 310–410 million NZ$ over five years (Greer and Saunders 2012). Large losses were due mainly to the complete susceptibility of the diploid *A. chinensis* yellow-fleshed kiwifruit cultivar ‘Hort16A’. In comparison, the green-fleshed *A. deliciosa* cultivar ‘Hayward’ shows a degree of resistance that allows the disease to be managed by orchard practices. Breeding for a Psa-resistant yellow-fleshed cultivar is a high priority for the kiwifruit industry in New Zealand. To this extent, evaluating parents and selecting those that show some degree of Psa resistance is an integral part of all current breeding programmes.

As *Actinidia* species are generally dioecious, progeny tests are the only way breeders can predict the breeding values for fruit traits of males. Factorial crossing designs are the norm, with about 25 full-sib female seedlings per individual cross planted for phenotypic evaluation. Tetraploid (4*x*) *A. chinensis* have been reportedly showing higher resistance to Psa than diploid (2*x*) populations (Gea et al. 2012; Montefiori 2013) and currently form the core population for breeding resistance to Psa in yellow-fleshed kiwifruit. A progeny trial involving crosses between four female and nineteen tetraploid male parents was planted in 2008. Following the Psa outbreak of 2010, these vines have been scored for disease severity and this data is the main focus of analyses in this study to understand the genetic architecture of resistance to Psa in kiwifruit.

Different methods and scales of measurement have been used to record the progress of Psa disease (Gea et al. 2012) at the individual vine level. Susceptibility to Psa does not show a clearly observable phenotypic progression. Thus, none of the different disease score monitoring scales has managed to record an ordinal progression of the disease through several scoring levels to enable the assumption to be made that the underlying scale is continuous. Quantitative genetic analyses that aim to estimate genetic parameters assume the scale of measurement to be continuous. Because of the non-ordinal nature of the disease-scoring scale in this study, we converted Psa score data to a binary scale (0 = no disease 1 = disease). Binary data are often presented as sample proportions for purposes of analyses. Issues of variance heterogeneity and non-normality in the sample proportions are traditionally handled using data transformations, such as angular transformation.

Estimation of variance components and best linear unbiased predictions (BLUPs) of genotype random effects on continuous traits by fitting linear mixed models (LMMs) to familial data is well established. Statistical models with complex variance structures that account for pedigree as well as spatial trends within a field layout have been extensively applied to such data to assess if the trait of interest has a significant genetic component and is heritable (Piepho et al. 2008). However, for transformed proportional data the use of LMMs can be limiting and results can be unreliable, particularly when sample sizes are variable and small. Furthermore, in case of some transformations such as the angular, model predictions back-transformed to the original proportional scale are not necessarily bounded in the interval [0, 1]. The empirical logit and probit transformations do not suffer from this problem. When estimating genetic parameters, such as the heritability, of binary traits, parameterisation is better handled on an underlying unbounded continuous liability scale in which it is most interpretable (Lee et al. 2011).

The generalized linear models (GLMs) of McCullagh and Nelder (1989) extended linear models (LM) to data that follow probability distributions other than Normal, but which still belong to the exponential family of distributions, such as the Poisson and binomial. The GLM applies only when the data are uncorrelated. The generalized linear mixed models (GLMMs) extend this by incorporating correlations among responses, which is accomplished by including random effects in the linear predictor and/or by modelling correlations directly (Schabenberger 2005). Piepho (1999) provide a good discussion with illustrated examples of the analyses of disease incidence data from designed experiments using GLMM. Originally developed for members of the exponential family, GLMMs have been extended to a much broader range of applications by using quasi-likelihood estimation methods (Littell et al. 2006). Thus, GLMMs are the logical choice for fitting variance components to binary familial data.

Our primary goal in this study was to conduct an in-depth analysis of a Psa progeny test dataset of tetraploid *A. chinensis* parents and provide reliable estimates of additive genetic and environmental variance components and narrow-sense heritability in relation to susceptibility to Psa. For reasons explained above, we used GLMM methodology applicable to binary/binomial distributed data. The fundamentals of GLMM were developed some time ago, but its implementations in widely available statistical software happened much later. The R lme4 package (Bates et al. 2014) was first uploaded in 2003, and the SAS® (SAS Institute Inc. 2013) Proc GLIMMIX became a standard procedure in V9.2 in 2008, although a production version was released in 2005. ASReml (Gilmour et al. 2009) is a specialised standalone software package for breeding data which uses the average information (AI) algorithm and sparse matrix methods for fitting LMMs. GenStat uses the same algorithm for its REML estimation. ASReml-R is the implementation of ASReml in R (Butler et al. 2009). While ASReml software can fit GLMMs, the fitting of GLMMs in ASReml-R appears to be limited. The GLIMMIX procedure in SAS® is becoming popular for fitting GLMMs in various disciplines and there are a few examples of its application in plant and animal breeding (Fikret 2011, Maxa et al. 2009). We have not come across applications of Proc GLIMMIX for progeny testing where pedigree information on parents is incorporated into the analysis. Unlike ASReml, Proc GLIMMIX is not specifically developed for analyses of breeding data; therefore some tweaking is necessary, depending on available data and analysis objectives. Our secondary goal, therefore, was to demonstrate the application of Proc GLIMMIX for fitting models that incorporate familial resemblances among and between parents and progeny, which is the case with the Psa progeny testing dataset presented in this study.

## Materials and methods

### Genetic material and field design

*Actinidia chinensis* taxa can be either diploid (2*x* = 58) or tetraploid (4*x* = 116). Families included in this study came from a factorial mating design where four female parents (labelled GU, GZ, GO & GT) were crossed to 19 male parents (numbered 28:46). This is the standard notation used in kiwifruit breeding in New Zealand, with females in a cross named by two letters, males by numerals and the family by concatenating the two labels. Some crosses were untried or unsuccessful: female GO, with the exception of crosses to males 32 and 33; male 32, apart from the cross to female GO; and female GU to male 33. For fitting the GLMM, we left out parents GO and 32 to provide a nearly balanced factorial crossing structure (3 females × 18 males) for the dataset with only one missing cross (GU × 33). Of the 55 full-sib families, 53 were retained in the subset that excluded one female and one male parent. All the parents used in crosses were tetraploid. The field experimentation was carried out at the Te Puke Research Centre in the Bay of Plenty (37.8°S 176.3°E), the major kiwifruit-growing region in New Zealand. On average about 36 seedlings from each full-sib family were field planted in spring (October) 2008 in a randomised block design with three replicates; each replicate comprised three consecutive rows except for the last replicate which had an additional row to accommodate extra seedlings of 8 families. The experimental layout, therefore, was not balanced. The family sizes varied from 24 to 50 depending on seed availability. The 10 rows in the experimental block ran in a south–north direction and contained posts spaced at 4 m within a row and with 3 m between rows. Progeny testing trials are usually planted at higher densities than commercial orchards. The experimental unit (plot) here was the bay between two posts within a row, where 12 unsexed seedlings of any one family were planted as a twin row, with seedlings alternating on either side of the trellis wire at six positions (a:f) placed at 60-cm intervals. There was a 40-cm distance between the seedlings in each twin row. A hedge of shelter trees was present on the west and south side about 4 m away from the experimental block. There were 19 bays (numbered 0:18) down each row of seedlings, with the first and last bays containing only two planting positions (a and b). Only half the seedlings planted were expected to be female, but both females and males can be assessed for Psa symptoms. Control plots of the *A. chinensis* cultivars ‘Hort16A’ and ‘Zesy003’ were included as end of row guards within the experiment.

**Figure 1:**
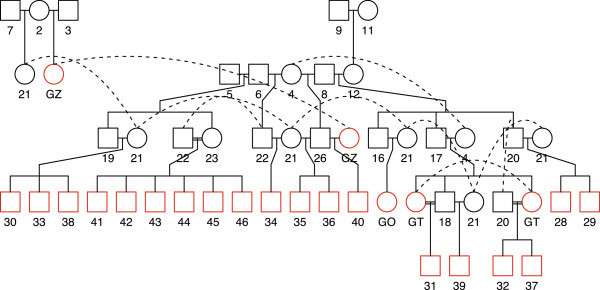
**Pedigree of** ***Actinidia chinensis*** **parents used in crosses to form full and half-sib progeny families.** Individuals with a coloured border are the parents used in this study (19 males and four females). The female parent 13 with unknown parentage is not included in the pedigree chart.

Figure 1 shows a pedigree diagram of the four female and 19 male parents generated by the R ‘kinship2’ package (Sinnwell et al. 2011). The coefficient of coancestry is a measure of the relatedness between two individuals that have a common ancestry (Falconer and Mackay 1996). In population genetics the genetic covariance between related individuals is written as a function of the relevant genetic variances weighted by a coefficient which reflects relatedness. The general formula for genetic covariance between two individuals *P* and *Q* in terms of the additive and dominance genetic variances is *Cov*(*P*, *Q*) = *rV*_*A*_ + *uV*_*D*_; and for diploids *r* = 2*f*_*PQ*_*u* = *f*_*AC*_*f*_*BD*_ + *f*_*AD*_*f*_*BC*_, where *f*_*PQ*_ is the coancestry of *P* and *Q* etc., and *A & B* and *C & D* are parents of *P* and *Q* respectively (Lynch and Walsh 1998). Considering the additive effects only, the coefficients *r*, computed pairwise, for a set of individuals form the additive or numerator relationship matrix **A**, which we used later to fit the GLMM. Given the pedigree records of parents and their progeny, we used the INBREED procedure in SAS to calculate **A**.

### Data

Psa symptoms were first detected in commercial kiwifruit orchards in Te Puke in November 2010. Psa is a systemic disease, with varying symptoms appearing in different parts of the vine as the disease progresses. This made it difficult to develop a single plant disease severity scale which was truly ordinal. During initial observations we recorded the key vine symptoms as: presence/absence of leaf spots, short dieback at the ends of the canes, cane dieback, cankers and oozing. Based on this, we defined a quasi-ordinal visual assessment scale (0 – 8) as follows: 0 = no symptoms, 1, 2, 3 = leaf spotting and damage at increasing intensity, 4 = oozing, with a qualifier to the score based on which plant part is involved: s – shoot, d - main leader and b – bud. 5, 6, 7, 8 = vine died and removed. The decision to remove plants with a Psa score higher than 4 was made to reduce inoculum levels and followed the recommendations of Kiwifruit Vine Health, the industry body charged with managing the disease. Monitoring the movement and progress of disease through the block was carried out fortnightly using the scale described, in 2010 and subsequent seasons. A preliminary analysis of the trends in disease score over time has been reported by Gea et al. (2013). For the genetic analysis that follows, we decided to take a point in time in monitoring when the disease had spread well within the block, but when there was still some variation in scores that would allow estimation of variability in disease score due to underlying environmental factors. On this basis, we selected for analysis the measurement of disease made in November 2012, i.e. 20 months after first detection in the research orchard. The non-ordinal nature of the measurement scale and the fact that a score of ≥ 4 triggered vine removal justified the data being converted into binary: 0 = score ≤ 3, and 1 otherwise. The threshold score agreed with our observations that if symptoms were restricted to leaf spots only, kiwifruit vines showed a degree of resistance that was deemed adequate to avoid systemic spread and vine death. Furthermore, this degree of resistance is probably sufficient for control of disease in the orchard by other management practices.

**Figure 2:**
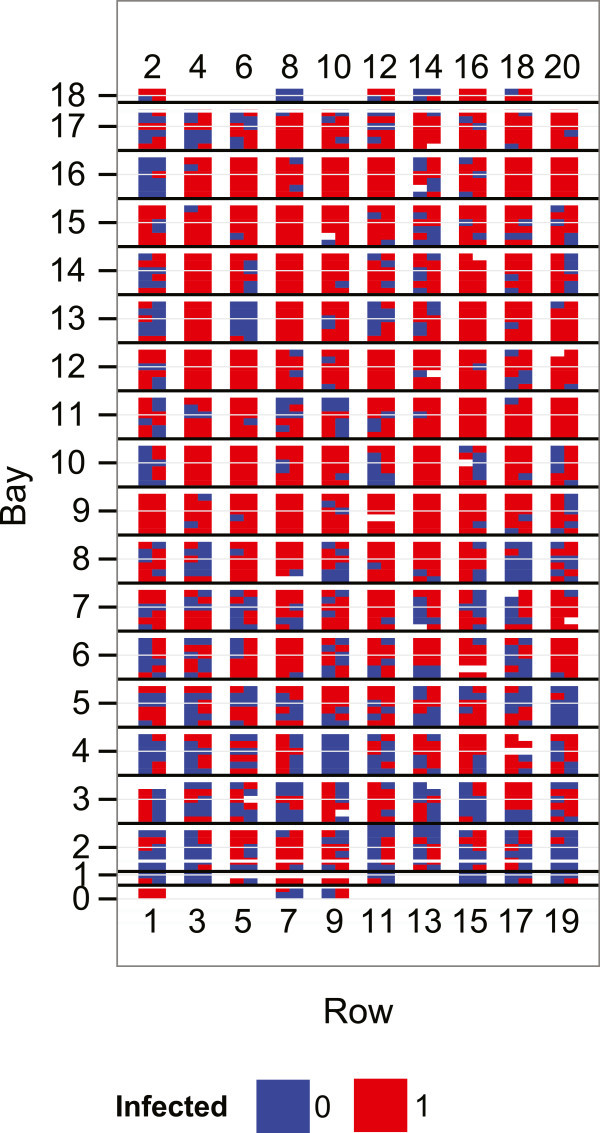
**Heatmap representing** ***Pseudomonas syringae*** **pv** ***. actinidiae*** **(Psa) scores of** ***Actinidia chinensis*** **(kiwifruit) seedlings, assessed in November 2012 and categorised as a binary outcome (present/absent), plotted on the field plan.** The figure is pointed upwards in north direction, and the hedges are located on the south and western sides of the plan.

A heatmap is a graphical representation of data values in a matrix by a colour scheme. Figure 2 shows a simple heatmap of the binary (0/1) disease outcomes constructed using the ggplot2 package (Wickham 2009) in R Core Team (2013). The heatmap illustrates the spatial variability in diseased (or healthy) vines across the rectangular experimental area at the beginning of November 2012. A hedge effect, which is common with other phenological and fruit traits, is also evident with disease incidence, with fewer diseased vines registered in the row closest to the hedge on the western side (left of figure) and in the more protected southern bays. Rows, and bays across rows, were two factors included when formulating the mixed model later on.

### Model specification and fitting

The generalised linear mixed model for Bernoulli or binomial outcomes is of the form
1

where ***y*** is the *n* × 1 vector of outcomes (0 or 1), *g*(.) is the link function which relates outcome ***y*** to the linear predictor ***η***, and *g*^− 1^(.) is the inverse of the link function. ***β*** and ***u*** are vectors of fixed and random effects respectively, with the corresponding design matrices **X** (*N* × *p*) and **Z** (*N* × *q*), and *y*_*i*_ is distributed according to any one of the exponential family of distributions. The GLIMMIX procedure distinguishes between random effects in the linear predictor (G-side) and/or modelling correlations among the data directly (R-side) (Schabenberger 2005). The two types of random effects specify the corresponding covariance structures ***G*** and ***R*** of the mixed model. It is assumed that random effects ***u*** have a normal distribution with mean **0** and variance matrix **G**, i.e. ***u*** ~ *N*(**0**, **G**). In the case of GLMM, the distribution of data ***y*** is specified conditional on random effects ***u***. The variance of **y** is a function of the mean for most distributions of the exponential family and is given by *Var*(***y***|***u***) = **A**^0.5^**RA**^0.5^, where **A** here is a diagonal matrix containing the variance functions (Schabenberger 2005) and **R** a working correlation matrix. By default, **R** is an identity matrix, i.e. with a scale parameter value equal to unity. In GLIMMIX, by including the statement RANDOM _RESIDUAL_, the procedure can be forced to estimate a dispersion parameter from the data.

For the Psa data, appropriate distributions in the case of individual seedling and plot outcomes are *y* ~ *Bernoulli* (*π*) and ∑ *y* ~ *Binomial* (*n*, *π*) respectively, where Pr(*y* = 1) = *π*. We used the logit link function in both instances, i.e. log_*e*_(*π*/(1 − *π*)). The following is a detailed specification of the GLMM used for Psa data where the response variable is binomial. As described earlier, Psa susceptibility scores were categorised as binary (0/1) at the vine unit level. As such, our observations for each plot (i.e. row x bay) consisted of the number of seedlings that were diseased out of *n* total seedlings. The sample sizes were small. Of the 169 plots, 149 had a full complement of *n* = 12 seedlings; for the remaining plots *n* varied from 4 – 10. The resulting linear model for the disease count *y*_*ij*(*klr*)_ of the plot at the *i*^*th*^ row and *j*^*th*^ bay (or column), with the notation indicating it is the *r*^*th*^ replicate sample plot of the family generated by crossing the *k*^*th*^ female parent with the *l*^*th*^ male, is:
2

where the row effect  and bay effect  captures the environmental variation in the response;  is the mean additive genetic effect of the *r*-th replicate full-sib sample of *kl*-th parents and **A** is the numerator relationship matrix between family samples;  is the family effect due to non-additive and other causes;  is the replicate block effect. All effects and corresponding variances are on the logit scale.

Appendix A shows how to fit the binomial model discussed in this paper using SAS®, and a brief description of the code is described below. The GLIMMIX procedure requires the additive random effects parameter variables of design matrix **Z** to be generated outside the procedure. For BLUP breeding value estimation in the plot aggregate analysis, the genetic units of interest are the family replicate samples of the progeny, and the parents. We fitted what is generally called the ‘animal model’ to estimate the additive genetic variance. Replicate plots of a given family are different samples of the same full-sib family; hence the resemblance between sample means, given the two parents, is that of full-sibs. Writing the SAS code to construct variables for additive random effects of **Z**, we first used the GLMMOD procedure to generate dummy variables *z*_*x* + 1_, …, *z*_*x* + *N*_,where *N* = number of plots or vines depending on the model fitted, and *x* = number of parents. Variables were numbered in the same order as plots were arranged in the phenotype dataset, i.e. bays within rows. Then, we added *x* additional variables (*z*_1_, …, *z*_*x*_) with all elements equal to zero to represent the additive random effects of parents who had no phenotypic values, but who needed their breeding values to be predicted by the model. The coefficients of the covariance matrix of random variables *z*_1_ − *z*_*x* + *y* + *N*_, where y = number of ancestors of the parents, is given by the additive relationship matrix **A**. This was constructed using Proc INBREED as discussed in a previous section, followed by manipulations as described below to form a SAS dataset we named ‘Adata’. For this we first updated the **A** by deleting from it the corresponding rows and columns for the *y* number of ancestors of parents for which we were not interested in estimating breeding values. Secondly, we added in two numeric column variables named PARM and ROW before columns Col1-Col(*x* + *N*) of the updated **A** to form ‘Adata’. Since we were interested only in the additive variance, PARM had a value = 1 for all rows in ‘Adata’, which were numbered 1 to *N* in the ROW variable. Now, the GLIMMIX procedure statement for estimating the additive genetic variance and breeding values has the syntax: RANDOM Z1-Z(*x* + *N*)/TYPE = LIN(1) LDATA = Adata SOLUTION. Other random effects shown in the specified model were simply coded by a separate RANDOM statement. A similar application in genomic selection using SAS Proc MIXED and its LIN(1) structure is given by Piepho et al. (2012).

Fitting GLMMs can be computationally challenging. The algorithm can fail to converge, particularly when the dataset is large and the model is complex. This is often the case with breeders’ field data where models can become complex because of the inclusion of many random effects with different types of covariance structures, such as those for familial resemblances and spatial correlations. In practice, compromises have to be made in the complexity of the specified model to achieve convergence. Proc GLIMMIX provides three types of residuals for diagnostic plots: raw, studentized and Pearson. Where random effects are involved, residuals can be obtained for the marginal or the conditional model. We used these plots to examine model assumptions and detect outliers.

### Genetic parameters and BLUPs

For each random effect included in the model, Proc GLIMMIX analysis provides an estimate of its variance component, and corresponding BLUP estimates for all levels of the factor. Estimated genetic and environmental variance components allow us to calculate heritability, which is a key genetic parameter of the trait. The problem with defining heritability in a consistent fashion is that different investigators may choose different selection units to which the estimated heritability is applicable. Hence, even with similar variance component estimates one could obtain different heritability values. In plant breeding, the selection unit can be an individual plant, a plot or even a whole family. In this study, we estimated heritability in the ‘narrow sense’, i.e. the variance due to additive genetic effects only, given as a proportion of the total phenotypic variance. The heritability on a plot basis was defined as:
3

where variance components apart from  are as described earlier and *n* is the harmonic mean of the seedling count per replicate plot (= ~10). Unlike linear models in GLMM, the variance is a function of the mean, and as such the scale parameter is set to unity and there is no separate estimate of residual variance from the fitted model. The default link function for binomial data is logit, hence the residual error variance  on the logit scale is usually taken as fixed and approximated by the variance of the standard logistic distribution, which is *π*^2^/3 (Gilmour et al. 1985). Binomial data can be over-dispersed, i.e. the estimated variance of the binomial random variable is greater than expected for the distribution. In such situations a scale parameter *φ* can be specified in the model such that *Var*(*y*) = *φ* × *Var*(*μ*), and the scale parameter is estimated from the data. If the scale parameter is significantly different from unity, the residual variance of the above equation should be set to .

As shown in Eq. 3, heritability is calculated as the ratio of two linear functions of estimated variance components. Together with variance parameters, Proc GLIMMIX outputs the estimated parameter estimate covariance matrix, which is needed for the estimation of standard errors of variance parameters and of heritability. The standard error of heritability can be calculated by the Delta method which uses approximations to the Taylor series expansion to compute the variance of functions of random variables. Details of the derivation and a SAS macro for the implementation of method are available in Appendix B.

The key BLUP values of interest in this study were the breeding values of the parents. The GLIMMIX procedure produces estimates of prediction variability for random effects  as , which is the variance of prediction error. Therefore, the standard error estimates of BLUPs presented here do not account for variability of the random effect parameter, *u*. The BLUP estimates output by analysis are on the liability scale (logit), which can be transformed to predicted probabilities after adding the estimate of the intercept,  and taking . The naive back-transformation though is not unbiased for *p*. The breeding values as such given as predicted probabilities to disease susceptibility were used to compare parents.

## Results

As seen in the pedigree (Figure 1), there were 19 ancestors of the 21 parents whose crosses were used in this analysis. Assuming that founder vines had no inbreeding, none of the female parents was inbred and of the 18 males (numbered 28–31 & 33–46), the male 31 had the highest degree of inbreeding (*F* = 0.25; its parents were full-sibs of unrelated ancestors 17 and 4). Male parent 37 descended from a cross between the male vine 20 and its brother’s (17) daughter (GT), which resulted in an *F* value of 0.125. Similarly, the male parents 41–46, which were full-sibs and whose parents (23 and 22) were half-sibs of the common female ancestor 4 (Figure 1), had an *F* value of 0.125. The remaining 10 males had no inbreeding. Relatedness between individuals as measured by the coancestry coefficient (*f*_*XY*_) ranged from 0 to 0.375 for the 21 parents. Female parent GU had no known relationship to any of the individuals in the pedigree (Figure 1). Similarly, GZ was unrelated to GT and males 31, 37 and 41–46; as were the pair of male full-sibs (28 and 29) unrelated to the set of full-sibs 41–46. All other combinations of parents had some relatedness, with GT and its progeny male 31 being the most related,  closely followed by the full-sib male parent set 41–46 at *f* = 0.3125. The INBREED procedure produced the additive relationship matrices (ARMs) for each of two levels of the dataset, i.e. individual vine and family plot mean. After truncation, the ARM for the 1882 phenotyped vines and their parents was of dimension 1903. Similarly, the 169 plot aggregates gave a matrix which was much smaller (190). Because the mating design was a factorial and the parents forming the crosses were not all unrelated, the ARMs constructed were reasonably dense with a large proportion of non-zero elements.

**Figure 3:**
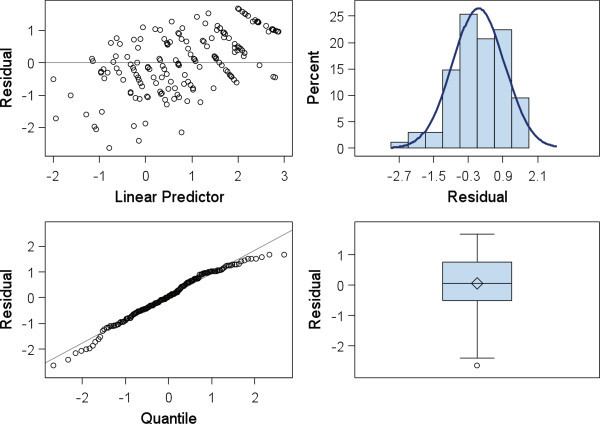
**Plots of conditional residuals of the fitted binomial generalised linear mixed model** (Eq. 2).

Given the relatedness matrices as calculated above, Proc GLIMMIX fitted the generalised mixed model as specified earlier to each of the individual and bay aggregate phenotypic datasets. The two models are hereafter called the Bernoulli and binomial, respectively. Both models estimated family × replicate interaction variance to be zero. Furthermore, the variance of the replicate effect was estimated as zero in the Bernoulli model, and very small and non-significant in the binomial model. Models were re-fitted by dropping out both these effects. Since rows were nested within blocks and both rows and bays were still in the model, we would not expect a loss of information on variation due to spatial effects in this reduced model. The GLIMMIX procedure code was implemented in SAS 9.4 (64 bit) installed on a laptop i5, 2.80 GHZ machine with 8 GB of RAM running the Windows 7 (64 bit) operating system. Both models achieved convergence; the binomial model took only 5.42 s user CPU time compared with the Bernoulli model with the much larger dataset, which took 13 h and 8 min. The GLIMMIX procedure provided information about the fitted model in terms of three statistics: − 2 Res Log Pseudo Likelihood, Generalized Chi Square and the ratio of the latter to its degrees of freedom. This ratio was close to 1, 0.80 and 0.92 respectively, for the Bernoulli and binomial, indicating no evidence for residual over-dispersion. Residuals are used to check if data meets the assumptions of the model and to detect any outliers and influential points. Plots of conditional studentized residuals of the binary model are shown in Figure 3. The BLUP estimator in mixed models has the property of shrinkage towards the mean; therefore the trend shown in the studentized residual v. linear predictor plot (Figure 3, top left panel) is as expected. There is no strong evidence of unusual observations, with only a few data points falling just outside the ± 2 limits commonly used as a rule of thumb for outlier detection. The conditional residuals are studentized and therefore should follow the standard normal distribution. The remaining plots in Figure 3 appear consistent with this assumption.

Variance components estimated by the Bernoulli and binomial models are presented in Table 1. These estimates, given on the logit scale, were similar for both models except for the family variance, which was more than twice as large and marginally significant in the Bernoulli model and non-significant in the binomial model. However, the family variance was low, particular when compared with the additive variance, which indicated that non-additive effects are not likely to be important in determining resistance to Psa. The variance component due to the row effect was slightly larger and marginally significant in the fitted Bernoulli model. Spatial variation in Psa within the experimental area, however, was largely due to the bay effect (Table 1), which was statistically significant in both models. This is consistent with what is seen in the heatmap (Figure 1), where higher numbered bays in general had more disease. The results presented from here onwards, unless stated otherwise, are for the fitted binomial model. When predicted bay random effects were ranked (1–19) from the smallest (lowest disease) to the largest, eight of the nine leading ranks fell in the bottom half (bays 0–8) in Figure 2. Similarly, the predicted random effect for row =1 was the smallest, indicating it had the lowest Psa disease among rows. It is well known from field observations that vines closer to the hedge are less likely to exhibit Psa disease. The tall hedge to the west (left in Figure 2) and south of the orchard block may have provided some protection to nearby vines. The predicted values for bays and rows were therefore consistent with what is known of the spatial variation in Psa within an orchard block.

**Table 1:** **Estimates of variance component parameters and the narrow-sense heritability obtained by fitting generalised linear mixed (GLMM) and linear mixed (LMM with empirical logit) models to** ***Pseudomonas syringae*** **pv** ***. actinidiae*** **(Psa) incidence in a set of factorial full-sib families of** ***Actinidia chinensis*** **seedlings**

Variance component	Binomial GLMM - SAS	Bernoulli GLMM -SAS	LMM - SAS	LMM - ASReml-R
Row,	0.074 ± 0.060	0.095 ± 0.061	0.053 ± 0.045	0.056 ± 0.006
Bay,	0.383 ± 0.169	0.375 ± 0.154	0.361 ± 0.149	0.358 ± 0.039
Family,	0.039 ± 0.096	0.108 ± 0.081	0.069 ± 0.072	0.075 ± 0.008
Additive,	0.687 ± 0.190	0.732 ± 0.201	1.359 ± 0.184	1.350 ± 0.147
Residual			4.5E-7 ± 3.3E-4	2.3e-4 ± 2.5e-5
Heritability,	0.57 ± 0.13	0.55 ± 0.11	0.74 ± 0.08	0.73

The models were fitted using the GLIMMIX and MIXED procedures in SAS® respectively, and in ASREml-R.

Narrow-sense heritability estimates for Psa susceptibility of the family means given on a logit scale as calculated by Eq. 3 are presented in Table 1. The values of narrow-sense heritability were moderate and very similar for the two models. In a similar genetic study on diploid *A. chinensis* populations, Cheng (2014) recently reported resistance measured by time-to-infection to be similarly heritable. In a naturally infected environment, binary scoring followed by modelling that incorporates spatial trends should minimize any confounding effects on the resistance measure itself and estimates derived thereafter caused by spatial variation within the field. Other researchers have also reported on inheritance of resistance to bacterial diseases in plants caused by other *Pseudomonas* spp. Olczak-Woltman et al. (2009) reported a broad-sense heritability estimate of 53% for resistance to angular leaf spot in cucumber caused by *P. syringae* pv. *lachrymans*. This estimate was based on resistance measured by disease scores made on F_2_ families. Similarly, Sthapit et al. (1995) presented broad-sense heritabilities ranging from 0.72 – 0.84 for sheath brown rot in rice caused by *P. fuscovaginae*, using F_3_ families of three crosses, and the analysis was based on% incidence. Resistance is a notion that can be measured only indirectly using different criteria, such as the% incidence and severity scores. Heritability estimates will vary depending on the type of measurement used as well as the scale of the response variable analysed. As discussed earlier, our estimate of heritability is presented on a logit liability scale.

Estimated breeding values of the three female and 18 male parents, on the scale of probability of Psa incidence, are presented in Table 2. The female parent GZ stands out as providing the most resistance through transmission of additive genetic effects to its progeny. The next in rank in terms of resistance were male parents 37 and 42 (Table 2) which were related with a coancestry value of 0.0625 (through the common ancestor 4, Figure 2), but were unrelated to the top ranked female GZ. This indicates that independent genetic sources for Psa resistance may exist among tetraploid *A. chinensis* genotypes. The most resistant of the 53 full-sib families was GZ37 which had only three diseased vines observed out of 48 vines in total across all replicate plots (6.25%). This family also had the largest predicted random effect for Psa resistance, which accounted for any non-additive gene effects on family performance. A prediction based on the average eBV of the two parents and the family random effect provided an estimate of 0.31 for Pr(Psa) for this family.

**Table 2:** **Estimated breeding values (eBV) of parents given by logit-backtransformed probabilities of** ***Pseudomonas syringae*** **pv** ***. actinidiae*** **(Psa) incidence predicted by the binomial generalised linear mixed models (GLMM) fitted to a set of factorial full-sib families of** ***Actinidia chinensis*** **seedlings**

	eBV of ***Pr*** (Psa)			
Parent ^a^	Mean	95% LCL	95% UCL	Rank
GU	0.89	0.79	0.95	20.5
GZ	0.26	0.15	0.41	1
GT	0.74	0.60	0.85	13
28	0.85	0.65	0.94	17
29	0.83	0.61	0.94	16
30	0.70	0.45	0.87	9.5
31	0.69	0.45	0.86	8
33	0.78	0.53	0.92	14
34	0.88	0.69	0.96	19
35	0.89	0.73	0.96	20.5
36	0.81	0.59	0.93	15
37	0.49	0.25	0.73	2.5
38	0.73	0.48	0.89	12
39	0.86	0.69	0.95	18
40	0.60	0.35	0.80	5
41	0.67	0.41	0.85	7
42	0.49	0.24	0.74	2.5
43	0.70	0.44	0.87	9.5
44	0.57	0.31	0.79	4
45	0.71	0.46	0.88	11
46	0.63	0.37	0.84	6

^a^Letter codes are for female and numerals for male vines.

## Discussion

The methods used in this study show that fitting GLMMs to binomial distributed data where disease status is categorised as 0/1 is a useful method to consider to obtain genetic parameter estimates for disease incidence. The GLIMMIX procedure in SAS is a useful tool for fitting GLMMs where the dataset is small to medium scale. As the scale grows, increasing memory is required and the CPU time can extend to many hours, as was evident when fitting the Bernoulli model in this study. Rescaling the data by aggregating counts at the plot level and using a binomial model instead made the convergence quicker, by as much as four orders of magnitude. We attempted to fit a GLMM that, in addition to the genetic covariance structure, included a spatial covariance. Spatial covariance structures can be specified in Proc GLIMMIX by the RANOM _RESIDUAL_ statement with options such as / TYPE = SP(EXP)(x y) where x and y here are the field coordinates for rows and bays. In principle one could also model spatial covariance on the G-side. The residual variance (R-side) would then correspond to a nugget effect. A detailed account of the application of spatial covariance in GLMMs with field experiments is given by Gotway and Stroup (1997). Unfortunately, a model that included both genetic and spatial covariances failed to converge. When the data are normally distributed, two other procedures in SAS can fit the simpler linear mixed models (LMMs): Proc MIXED and Proc HPMIXED. The HPMIXED procedure uses sparse matrix algorithms and is particularly useful for large-scale datasets, but lacks the option for a linear coefficient matrix, needed for specifying an additive covariance structure in BV estimation. The default estimation method in Proc GLIMMIX for models containing random effects is known as residual pseudo likelihood (Wolfinger and O’Connell 1993) and, as the name suggests, it is computed from the pseudo- rather than the true-likelihood (Schabenberger 2005; Littell et al. 2006).

Our analyses show, the use of GLMMs is not fool-proof, and there are several potential pitfalls. The failure to account for overdispersion is probably the most common problem. Convergence problems may be an issue and furthermore particularly with binary data GLMM fits may be severely biased, particularly when sample size *n* is small. A detailed analysis of the bias problem is given by Engel (1998) and a good practical review and discussion of bias and other problems in the application of GLMM is given by Bolker et al. (2009). As the latter paper points out for a GLMM to get maximum likelihood (ML) estimates, one must integrate the likelihood over all possible values of the random effects and this calculation is at best slow, and at worst computationally infeasible. The same authors point out that for these reasons statisticians have proposed various ways to approximate the likelihood and estimate GLMM parameters and these include pseudo- and penalised quasilikelihood (PQL), Laplace approximations, Gauss-Hermite quadrature (GHQ) and Markov chain Monte Carlo (Hadfield 2010) algorithms. The PQL algorithm, which we used in our analysis, is the simplest and most widely used approximation (Bolker et al. 2009). The psuedo likelihood algorithm, however, is prone to biases when the binomial sample sizes (*n*) are small. Gauss quadrature is more accurate to compute the likelihood but slower and can only be used with GLMM having a single random effect and hence could not help for the models considered in this study.

Given the possibility of substantial bias in GLMM parameter estimates, as an alternative approach for comparison we fitted our model as a LMM using an empirical logit transformation of the proportional data. Piepho (2003) provides a good discussion of available transformations for proportional data. Except for the angular, most transformations fail when an observed proportion is 0 or 1, but some functions may be modified to account for such. We used the empirical logit transformation, log((*x* + *c*)/(1 − *x* + *c*)) (Atkinson 1985), where *x* is the observed proportion and *c* a small constant = 0.5/*n* when the data are binomial. The model was fitted using Proc MIXED in SAS® as well as in ASReml-R. Comparison of variance component estimates between GLMM and LMM (Table 1) appear to suggest that former underestimated the additive variance for which the random effects had a complex covariance structure, The sum of variance component estimates in the LMM (Table 1) agrees better with the total sample variance value of 2.04 for the empirical logit transformed data. The narrow-sense heritabilities estimated by LMM were much higher than those of GLMMs (Table 1).

In a GLMM framework, the definition of heritability of a binomial trait on the latent scale is problematic. The heritability equation (3) implies that one can compute a “phenotype” on the latent scale which is not the case. So, any such heritability estimate for GLMM (Table 1) is somewhat hypothetical. In a recent application of GLMMs with binomial data using ASReml 3.0 (Gilmour et al. 2009), Bennewitz et al. (2014) discuss the issues in heritability estimation for binary traits. As pointed out in the paper, the challenge in defining heritability using GLMM is that part of the nongenetic variation occurs on the observed scale, whereas the genetic effects occur in the link scale. These authors proposed an approximation for residual variance given as a function of the values of linear predictor, overdispersion parameter and the sample size, *n*. Once calculated, the individual residual variances are averaged across all subjects and used in the heritability equation.

Polyploid genetics is complex, with pairing patterns varying from polysomy to preferential to disomy and the possibility of double reduction in allele segregation, Estimators of even common population parameters become difficult to derive and often have to rely on large assumptions compared with those needed when dealing with diploids (De Silva et al. 2005; De Silva et al. 2006). In this study of tetraploid *A. chinensis*, our calculation of coancestry coefficients and estimation of the additive genetic variance component thereafter were based on the assumption of diploid inheritance. Autopolyploids such as tetraploid potato generally show polysomic inheritance compared with disomic inheritance in diploids and most allopolyploids. Even in allopolyploids, preferential pairing may not be perfect and some multivalent pairing is possible, which often leads to distortions in allele segregation. Recently, a detailed investigation of the pairing behaviour of tetraploid *A. chinensis* has been reported by Wu et al. (2014). Where pedigree information is not available, molecular markers can be used to estimate pair-wise relatedness coefficients, and suitable statistical methods are well developed for diploids (Ritland 1996; Lynch and Ritland 1999). More recently, these estimators have been extended to cover tetraploids, where the relatedness coefficient has been expressed as:  (Huang et al. 2014), where *Δ*_*i*_ is the probability that at any given locus, two tetraploid individuals share *i* alleles identical-by-descent. This and the derivations made by Kerr et al. (2012) suggest that, in the absence of double reduction, *r* is independent of the ploidy level. From a practical point of view this implies that the use of the additive (**A**) matrix as calculated in this study for genetic analysis of tetraploids should not give substantial bias in the additive variance estimate.

Heritability is a key parameter in quantitative genetics because it determines the response to selection (Piepho and Möhring 2007). The results of this study that showed a moderate heritability for Psa resistance suggest that selection for Psa tolerance is possible and that the genes involved are available in the Plant & Food Research (PFR) *A. chinensis* breeding populations. The implications are relevant to the choice of methodology used to select robust parents with Psa tolerance. The ability to correlate bioassay tolerance (Hoyte et al. 2013) with field performance will ensure speed and accuracy for disease resistance screening and breeding of new cultivars. Carefully planned mating designs will be needed to minimise inbreeding in order to sustain a long-term breeding strategy, as the number of tolerant parents is not large and Psa tolerance is just one of many important attributes needed for the selection of new cultivars.

### Ethical standards

The authors declare that the experiments comply with current laws of the country in which they were performed.

## Appendix A

SAS® code for fitting the generalised linear mixed models (GLMM) described in this paper.

## Appendix B

Calculating the **s**tandard error of heritability using delta method.

Let *f* = *u*/*v*, then the variance of the first order approximation of *f*(.) about their expected values *μ*_*u*_ and *μ*_*v*_ is given by Lynch and Walsh (1998).
4

Now let  = the estimated variance component vector, (***l***_***u***_, ***l***_***v***_) = the vectors of coefficients of the numerator and denominator in the heritability equation, and  = the parameter estimate covariance matrix. Then, , and () = (). These estimated values are substituted in Eq. 4 to give the variance of heritability. A SAS macro that implements the method is given below:
